# In vitro model of retinoblastoma derived tumor and stromal cells for tumor microenvironment (TME) studies

**DOI:** 10.1038/s41419-024-07285-2

**Published:** 2024-12-18

**Authors:** Emily Alefeld, André Haase, Dario Van Meenen, Bettina Budeus, Oliver Dräger, Natalia Miroschnikov, Saskia Ting, Deniz Kanber, Eva Biewald, Nikolaos Bechrakis, Nicole Dünker, Maike Anna Busch

**Affiliations:** 1 Institute for Anatomy II, Department of Neuroanatomy, Center for Translational Neuro- and Behavioral Sciences (C-TNBS), University of Duisburg-Essen, Medical Faculty, Essen, Germany; 2 Institute for Cell Biology, University Hospital Essen, Essen, Germany; 3 Institute of Cellular Neurophysiology, Medical Faculty, University of Bielefeld, Bielefeld, Germany; 4 Institute of Pathology Nordhessen, Kassel, Germany; 5 Institute of Human Genetics, Medical Faculty, University of Duisburg-Essen, Essen, Germany; 6 Department of Ophthalmology, Medical Faculty, University of Duisburg-Essen, Essen, Germany

**Keywords:** Preclinical research, Cancer microenvironment, Mechanisms of disease, Eye cancer, Cancer in the nervous system

## Abstract

Retinoblastoma (RB) is an intraocular tumor arising from retinal cone progenitor cells affecting young children. In the last couple of years, RB treatment evolved towards eye preserving therapies. Therefore, investigating intratumoral differences and the RB tumor microenvironment (TME), regulating tumorigenesis and metastasis, is crucial. How RB cells and their TME are involved in tumor development needs to be elucidated using in vitro models including RB derived stromal cells. In the study presented, we established primary RB derived tumor and stromal cell cultures and compared them by RNAseq analysis to identify their gene expression signatures. RB tumor cells cultivated in serum containing medium were more differentiated compared to RB tumor cells grown in serum-free medium displaying a stem cell like phenotype. In addition, we identified differentially expressed genes for RB tumor and stromal derived cells. Furthermore, we immortalized cells of a *RB1* mutated, MYCN amplified and trefoil factor family peptid 1 (TFF1) positive RB tumor and RB derived non-tumor stromal tissue. We characterized both immortalized cell lines using a human oncology proteome array, immunofluorescence staining of different markers and in vitro cell growth analyses. Tumor formation of the immortalized RB tumor cell line was investigated in a chicken chorioallantoic membrane (CAM) model. Our studies revealed that the RB stromal derived cell line comprises tumor associated macrophages (TAMs), glia and cancer associated fibroblasts (CAFs), we were able to successfully separate via magnetic cell separation (MACS). For co-cultivation studies, we established a 3D spheroid model with RB tumor and RB derived stromal cells. In summary, we established an in vitro model system to investigate the interaction of RB tumor cells with their TME. Our findings contribute to a better understanding of the relationship between RB tumor malignancy and its TME and will facilitate the development of effective treatment options for eye preserving therapies.

## Introduction

Retinoblastoma (RB) is the most common pediatric eye cancer, originating from cone precursors of the immature retina and is characterized by uncontrolled proliferation [1]. Retinoblastoma belongs to the six index childhood tumors proposed by the World Health Organization Global Initiative. In western countries, high cure rates are achieved due to advanced treatment opportunities involving chemo-, cryo- and brachytherapy as well as external beam irradiation and enucleation [2]. However, 50% of the children living in middle- and low-income countries die because of metastatic spread of the disease [3].

RB is initiated by the inactivation of the tumor suppressor gene *RB1*, however, different genomic alterations are involved in the metastatic disease (for review see: [1]). A recent study classified two RB subtypes [4] displaying different molecular and histological features, with RB subtype 2 tumors showing higher metastatic potential, expressing the recently identified marker trefoil factor family peptid 1 (TFF1) [4–8] and harboring *MYCN* gene amplifications [4].

The development of preclinical retinoblastoma tumor models is urgently needed to decipher the molecular mechanisms of RB pathogenesis, offering a platform for exploring potential therapeutic targets and testing novel treatment modalities to increase the overall survival of the patients. Several xenograft models and primary cell cultures have been established [9–13], however, only a few long-term cultures of RB cell lines are available [14–16], which are supposed to be genetically related to each other [17]. Moreover, several genetically engineered mouse models with conditional inactivation of *Rb1* and constitutive *p107* or *p130* loss have been established [18–21] as well as a murine model of *MYCN* amplified RB [22]. In addition, a human patient-derived iPSC (induced pluripotent stem cell) organoid model with germline cancer predisposing mutations has recently been published [23] as well as an organoid model established from fresh tumor material showing the existence of glia cells [24]. However, both systems have limitations, e.g. species-specific differences between mouse and human as well as inefficient and time-consuming tumor development. Of notice, the significance of investigating the tumor microenvironment (TME) cannot be overstated in RB related research as recent studies showed that the TME is involved in the regulation of tumorigenesis and metastasis of several tumor entities [25, 26]. Investigating how retinoblastoma cells interact with their TME would provide critical insights into the factors influencing RB tumor growth, invasion, and response to therapeutic interventions. Previous studies immunohistochemically revealed the existence of stromal cell types in the RB TME [27] and showed that proliferation of cone-like RB cells is induced by IGFBP-5 from retinal astrocytes [28]. In addition, some studies revealed that macrophages are critical factors during RB tumor progression in mice [29] and create an immunosuppressive environment [30]. However, it is still unknown how tumor and stromal cells interact during malignant progression of RB. In this regard, the development and characterization of long-term RB tumor cell lines and RB derived stromal cell cultures are essential as model systems to investigate their interaction in a tumor scenario closer to the in vivo situation.

In the study presented, we established and characterized primary RB tumor cells and RB derived stromal cells cultured under different growth conditions in order to identify individual characteristics of both groups. In addition, we successfully immortalized primary RB tumor cells and RB derived stromal cells and established 3D spheroid cultures, creating long-term cell cultures models to investigate RB tumor biology as well as the interaction of RB tumor and stroma to gain a deeper insight into tumor development and progression for future therapy optimization.

## Results

### Establishment of primary retinoblastoma cell cultures

Patients‘ cohort (Table 1) consisted of two males and three females with a mean age of 18 month. One patient’s tumor (T4) was classified as RB subtype 1 without TFF1 expression and invasion of the choroid or optic nerve. Three patients (T11, T14 and T18) displayed tumors of RB subtype 2 with TFF1 expression and at least invasion into the optic nerve. One patient’s tumor (T7) presented a partial TFF1 expression in immunohistochemically staining, without TFF1 secretion into the aqueous humor, and no invasion into the choroid or optic nerve. Therefore, a distinct assignment to a specific RB subtype was difficult.

**Table 1:** Histopathological features of RB tumors used in the study.

Patient	T4	T7	T11	T14	T18
**Age [month]**	6	28	11	24	21
**Sex**	male	female	male	female	female
**Laterality**	UL	UL	UL	UL	UL
**ICRB group**	E	E	E	E	E
**Chemo**	NC	NC	NC	NC	NC
***RB1*** **mutation [RB tumor tissue and tumor cell lines]**	c.2359 C > T IVS12 + 1 G > A	c.2371 A > T LOH	no mutation found	homozygous deletion	complex rearragement with partial LOH and dosis alteration
**Histopathological features**	**T4**	**T7**	**T11**	**T14**	**T18**
Invasion of choroid	no	no	yes	no	no
Invasion of optic nerve	no	no	yes	yes	yes
**TFF1 expression**	**T4**	**T7**	**T11**	**T14**	**T18**
Tumor IHC	no	partial	yes	yes	yes
NGS analysis	no	low	moderat	high	high
Aqueous humor	ND	no	no	yes	yes
Cell culture supernatant	ND	no	yes	yes	yes
**RB subgroup**	1	1 or 2	2	2	2

*UL* unilateral, *NC* no chemotherapy, *RB* retinoblastoma, *ICRB* International Classification of Retinoblastoma, *ND* not done, *NA* not analyzed, *MYCN* N-myc proto-oncogene protein, *amp* amplification.

**Fig. 1:**
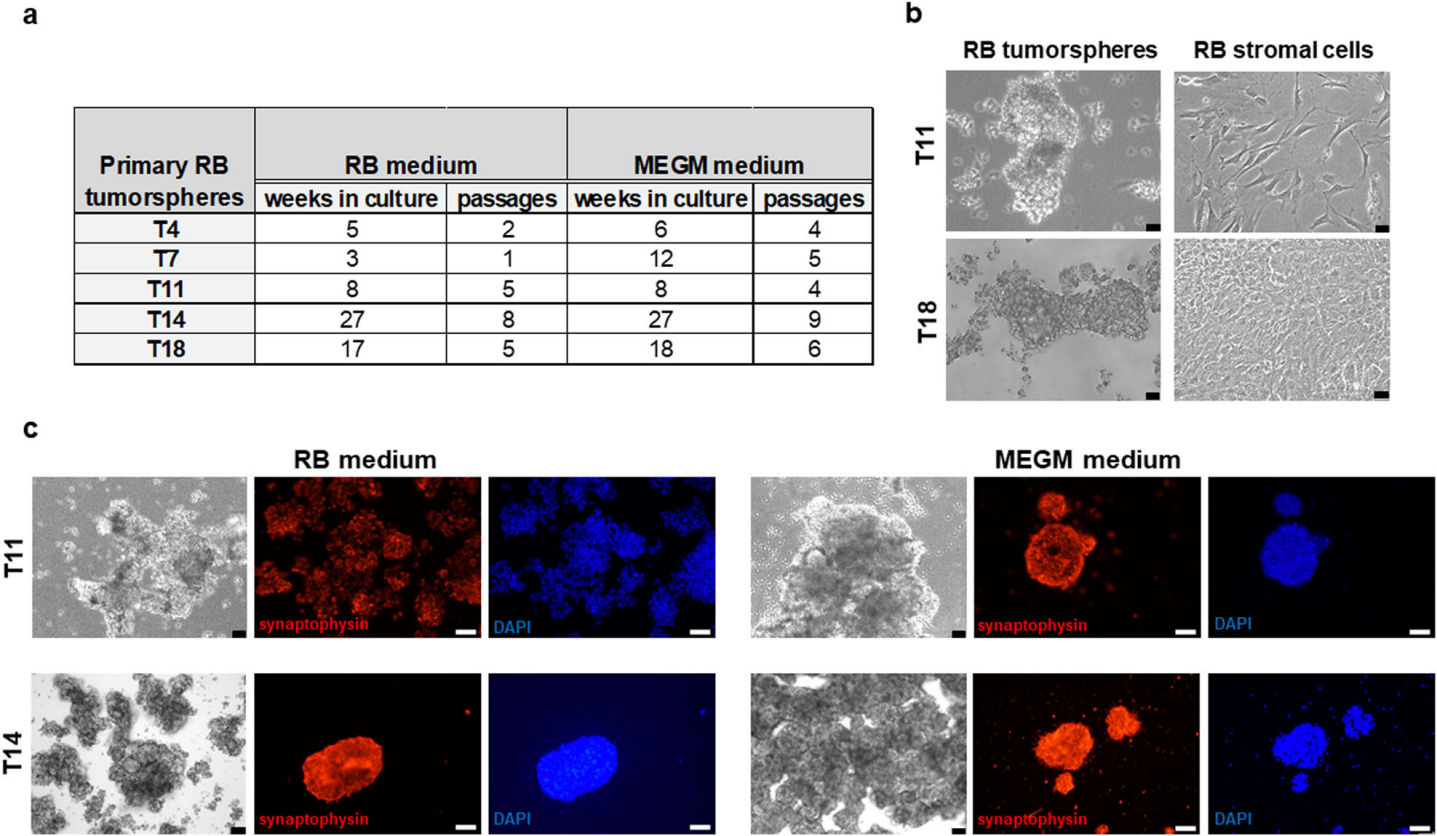
Overview and comparison of primary RB tumorspheres cultured in RB and MEGM medium. **a** Table summarizing weeks in culture and cell culture passages of the different RB tumorsphere specimens (T4, T7, T11, T14, T18) in the two different culture media used (RB and MEGM medium). **b** Phase contrast microscopy photographs comparing the morphology of unselected RB derived tumorspheres and stromal derived RB cells derived from two primary RB tumors (T11 and T18) cultured in RB medium. (scale: 64 µm). **c** Phase contrast imaging (black and white photos; scale bars: 64 µm) and immunofluorescence staining of two primary RB derived tumorsphere cell cultures (T11 and T14) maintained in two different growth media (RB medium in the left panel; MEGM medium in the right panel) with an antibody against synaptophysin (red fluorescence), a neuronal tumor marker and counterstained with DAPI (blue fluorescence; scale bars: 50 µm).

We established primary RB cell cultures from five patients (Table 1) in two media (RB and MEGM). All RB tumors formed proliferative tumorspheres with different growth behaviors in both media (Fig. 1a). Three RB tumors (T4, T11, T18) cultured in serum-containing RB medium formed an attached stroma-like monolayer, not seen in MEGM medium (Fig. 1b). To determine the origin of these stroma-like cells, we analyzed their RB1 mutation status. Tumorsphere cultures showed the original tumor’s *RB1* mutation, while stroma-like cells were *RB1* wildtype. Tumorspheres in both media were positive for the neural marker synaptophysin (Fig. 1c) and RB subtype 2 marker TFF1 [7].

### RNAseq analysis revealed differing gene expression patterns for RB tumorspheres cultured in different growth media

**Fig. 2:**
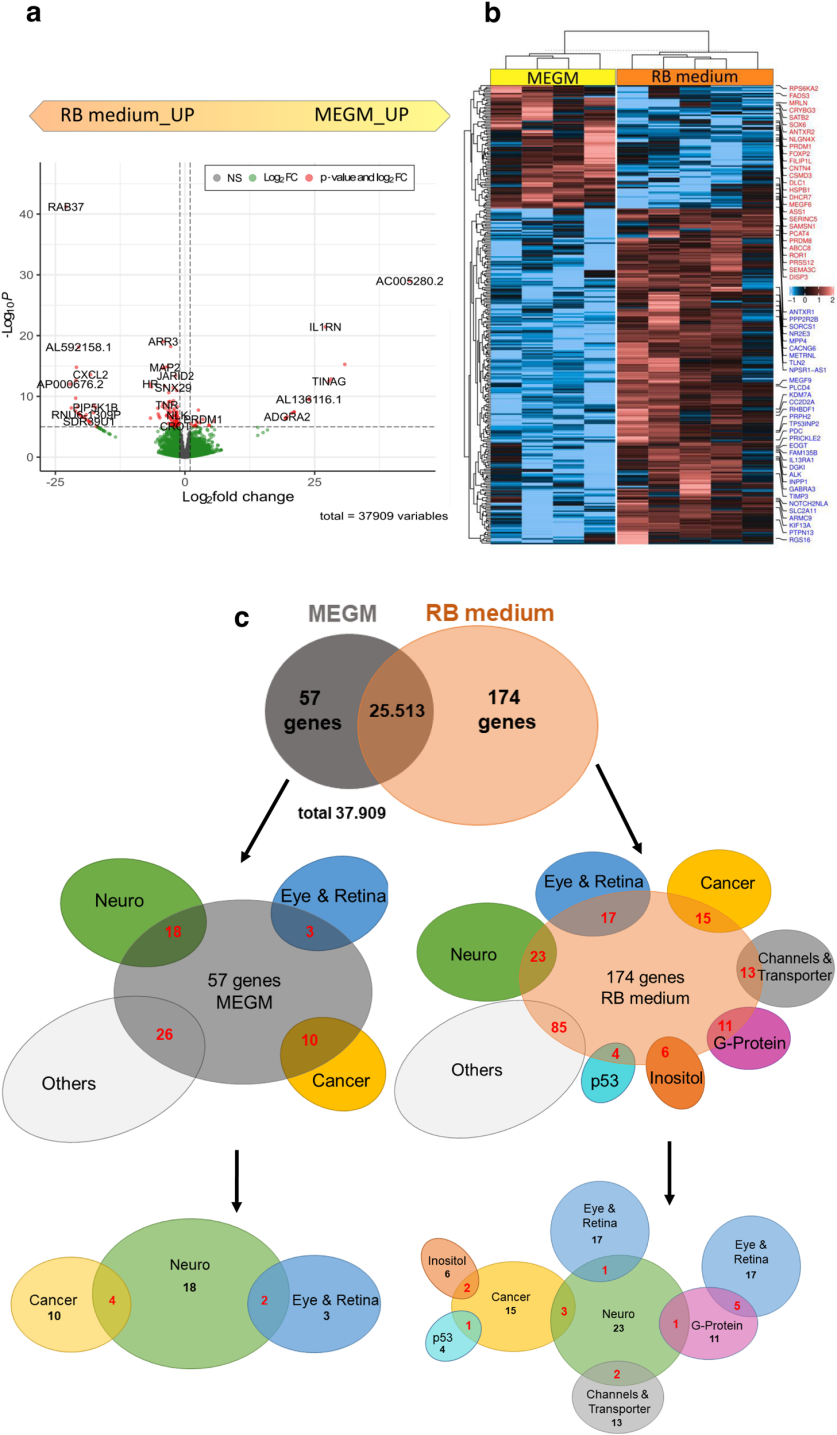
RNAseq analysis of primary RB tumorspheres cultured in RB and MEGM medium. Volcano plot (**a**), heatmap (**b**) and Venn diagram (**c**) of differentially expressed genes (DEGs) of primary RB tumorspheres either grown in RB or MEGM media as revealed by RNAseq analysis. **a** Significantly upregulated DEGs are depicted in red (*P*-Value > 0.05); upregulated DEGs are depicted in green (Log_2_ FC); non-regulated genes are depicted in grey. **b** Heatmap of significantly up- and down-regulated DEGs with a minimum fold change of 1.5 in all cell lines analyzed, the highlighted genes deviates in individual specimens are shown in red (MEGM group) and blue (RB group). **c** Venn diagram analysis of 231 DEGs identified comparing RB tumorspheres grown in MEGM or RB media. The 57 DEGs of the MEGM group and the 174 DEGs of the RB group were categorized into functional groups comprising of at least three DEGs of one RB relevant topic (red numbers in middle row of blots). The lowermost blots depict functional groups connected to each and in the overlapping areas, numbers of DEGs belonging to more than one RB relevant topic is displayed (red numbers).

To gain a deeper insight into potential molecular differences, we analyzed and compared gene expression profiles of tumorspheres grown in RB medium (T4, T7, T11, T14, T18) with those grown in MEGM medium (T7, T11, T14, T18) using RNAseq analysis, covering 37,909 genes. Differential expressed genes (DEGs) between the two groups (RB vs MEGM) were selected by setting a minimum *P*-value of 0.05 and a 1.5 fold change (FC) as a cut off. We identified 231 DEGs between the two groups, 174 of them being upregulated in the RB group and 57 being overexpressed in the MEGM group (Fig. 2). Thereof, 27 of 57 DEGs overexpressed in the MEGM and 139 of 174 DEGs in the RB group are universally expressed across all samples analyzed. The expression of the remaining genes deviates between individual specimen, most frequently in T18 compared to the others (Fig. 2b).

### Categorization of the identified DEGs

Next, we performed a literature-based categorization of the identified DEGs into groups representing recurrent relevant topics for RB (Fig. 2c). MEGM DEGs are mainly associated with the topics ‘cancer’, ‘neuro’ and ‘eye & retina’ or two combined topics like ‘cancer and neuro’ (SEMA3C, ROR1, EPHA7, GDNFR) or ‘eye & retina and neuro’ (PRDM8, CRYBG3). One third of the MEGM DEGs classified as the ‘neuro’ group are connected to neuronal developmental processes or are expressed in the fetal brain (CSMD3, SEMA3D, UNC5C, FOXP2, SOX6, CRYBG3).

Relevant topics connected to RB DEGs are ‘cancer’, ‘neuro’, ‘eye & retina’ as well as ‘transporter’, ‘G-protein’, ‘inositol’ and ‘p53’ resulting in a more complex network of intergroup overlaps shown in Fig. 2c. Notably, one third of the DEGs classified as the ‘eye & retina’ category are either directly connected to retinoblastoma (NR2E3, RS1, PRPH2) or cone markers (PDE6H, ARR3, HRASLS) and 45% of the DEGs in the ‘eye & retina’ category also belong to the ‘G-protein’ group of genes (ARR3, PDC, RGS16, GNGT2, RGS9). Three genes from the ‘cancer’ and ‘neuro’ group are overlapping (ALK, TLX2, THY1), with THY1 being also directly connected to retinoblastoma [31, 32]. Seventy percent of the fourth largest group ‘channels & transporter’ includes genes involved in sodium/potassium-dependent transport mechanisms. The DEG in the ‘inositol’ group with the highest fold change is INPP4B, recently discovered by our group as a tumor suppressor gene in chemotherapy-resistant RBs [33]. In addition, one gene (LINC-PINT) from the ‘p53’ group of genes (TP53I3, TP53INP2, HIPK2) is likewise directly connected to retinoblastoma [34].

### Pathway enrichment analyses of the identified DEGs

A pathway enrichment analysis (DAVID analyses) of the identified DEGs revealed several GO terms and KEGG pathways with *P* < 0.05 in both groups (Supplementary table 3). Significant GO terms of DEGs belonging to the RB medium group refer to ‘biological processes’ as e.g., ‘visual perception’, ‘phototransduction’ or ‘retina layer formation’ as well as ‘regulation of G-protein coupled receptor protein signaling pathway’. The combination of the GO term ‘cell composition’ with the term ‘photoreceptor inner and outer segment’ hints to more mature or at least further differentiated characteristics of this group. In addition, the GO term ‘molecular function’ and the KEGG pathway analysis revealed a highly significant involvement of ‘ion channel binding’ and the ‘phosphatidylinisitol signaling system’ in the RB DEG group of genes. Significant GO terms of ‘biological processes’ of the MEGM group of DEGs are e.g., ‘brain development’, ‘neuron projection development’ and ‘axon guidance’ reflecting more immature, early developmental characteristics of this group.

Combined, both analyses identified RB cells grown in RB medium as more differentiated and specialized, whereas RB cells grown in MEGM medium are characterized by less mature, early developmental features.

### Identification of differential gene expression patterns of RB tumorspheres and RB derived stromal cells by RNAseq analysis

**Fig. 3:**
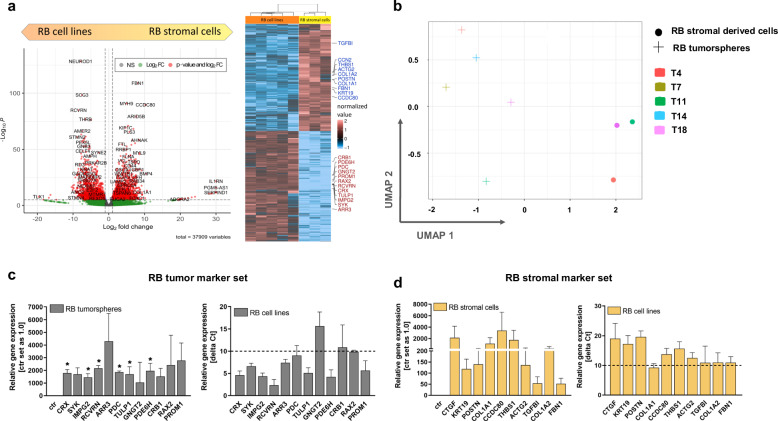
Volcano plot, heatmap and UMAP analysis and endogenous expression levels of identified marker sets for RB tumorspheres and RB derived stromal cells grown in RB medium. **a** Significantly upregulated DEGs are depicted in red (*P*-Value > 0.05); upregulated DEGs are depicted in green (Log2 FC); non-regulated genes are depicted in grey (volcano plot). Heatmap of significantly up- and down-regulated DEGs with a minimum fold change of 1.5 and highlighted genes of the “stromal marker set” in red and “RB marker set” in blue. **b** UMAP analysis of stroma-like RB cells (T4, T11 and T18) and RB tumorspheres (T4, 7, 11, 14 and 18) derived from five different patients displayed a clear separation of both groups. **c**, **d** Relative expression levels of genes defined as “RB marker set” in RB tumorspheres in comparison to the respective stromal cells (ctr) of the same specimen (**c**) and relative expression levels of genes defined as “stromal marker set” in RB derived stromal cells in comparison to the RB tumorspheres (ctr) of the same specimen (**d**) as revealed by real-time PCR. Expression levels of both gene marker sets were additionally analyzed by real-time PCR in four RB cells lines (Y79, WERI-Rb1, RB355 and Rbl-13; right graph in **c,** **d**). Delta Ct values are shown with a delta Ct of 10 equating a Ct value > 30, being defined as not expressed (**c**, **d**). Values are means of three independent RB tumor specimens or four independent RB cell lines ± SEM. *P < 0.05 statistical differences compared to the control group calculated by Student’s *t*-test.

To identify DEGs comparing RB tumorsphere and RB derived stromal cells we selected DEGs with a minimum *P*-value of 0.05 and a 1.5 FC as cut off. We identified 5373 genes differentially expressed in the two groups, 3077 DEGs being upregulated in RB tumorspheres and 2,296 DEGs being upregulated in RB stromal derived cells (Fig. 3a). However, 20,312 genes were not differentially expressed in either group. Uniform manifold approximation and projection for dimension reduction (UMAP) analysis revealed a clear separation of the stromal derived RB group compared to the RB tumorsphere group (Fig. 3b). Pathway enrichment analysis were performed to identify related gene functions of the DEGs. DAVID analyses revealed several GO terms with *P* < 0.05 in both groups (Supplementary table 4). While the GO terms ‘biological processes’, ‘cell compositions’ and ‘molecular functions’ revealed more general terms like ‘cell adhesion’, ‘cell surface’ or ‘protein binding’ in the stroma derived group, the GO terms in the RB group are highly specific for terms like ‘visual perception’, ‘photoreceptor inner and outer segment’ and ‘voltage gated potassium channel activity’. In addition, three significant eye-related diseases are connected to the RB tumorsphere group (Supplementary table 4).

These results show a clear separation of RB tumor and stromal components in the established primary cell cultures.

### Verification of RB stromal and tumor marker gene sets by real-time PCR

In order to verify next generation sequencing (NGS) data and identify a specific gene expression marker set for RB tumor and stromal cells, we analyzed up to 12 DEGs for each group by real-time PCR (Fig. 3c, d). To reduce individual heterogeneity, we compared three RB tumorspheres with RB stromal cells from the same respective patients (T4, T11 and T18). The analyzed genes were specific for each group (Fig. 3c, d), however, without expression levels reaching significance in the RB stromal derived group due to high inter-individual differences in the three specimens analyzed. We additionally verified the expression of the identified marker sets in four RB tumor cell lines (Y79, WERI-Rb1, RB355 and Rbl-13), showing that all identified RB tumorsphere markers, except for GNGT2, CRB1 and RAX2, were also expressed in the RB tumor cell lines, whereas the stromal markers were not expressed (Fig. 3c, d; delta Ct higher than 10 equates to Ct value > 30, defined as not expressed).

Thus, separate marker panels for RB tumor and RB stromal cells were established for further TME investigations.

### Comparison of primary RB cells with the original RB tumor

To compare the established primary tumor cells in MEGM and RB medium as well as the RB-derived stromal cells with the tissue of origin, the original T18 tumor was exemplarily sequenced. UMAP analysis revealed a separation of RB-derived stromal cells from the original tumor and primary tumor cells (Supplementary Fig. 1). To identify DEGs, we compared the original tumor with the primary RB cell lines cultured in the two different media and the stromal derived cells and set a 1.5 FC as a cut-off. We could show that the original tumor and both primary RB cell lines (RB and MEGM) clustered together with only one DEG identified in each group (EGR1 in the MEGM and NPVF in the RB group). By contrast, 758 DEGs were identified in the RB-derived stromal cell group compared to the original tumor and the primary RB tumor cells (Supplementary Fig. 1).

The results further strengthen the notion that the established RB tumor cells are closely related to the tumor of origin and that the RB-derived stromal cells are distinct.

### Immortalization and comparison of RB tumorsphere and stroma-like cells

Establishing long-term cell cultures would provide an opportunity to investigate the interaction between RB tumor cells and the TME. As to our knowledge, so far no RB derived non-tumor cell line for conducting such investigations is available, we set out to immortalize not only the primary RB tumorspheres, but also the RB tumor derived stromal cells.

**Fig. 4:**
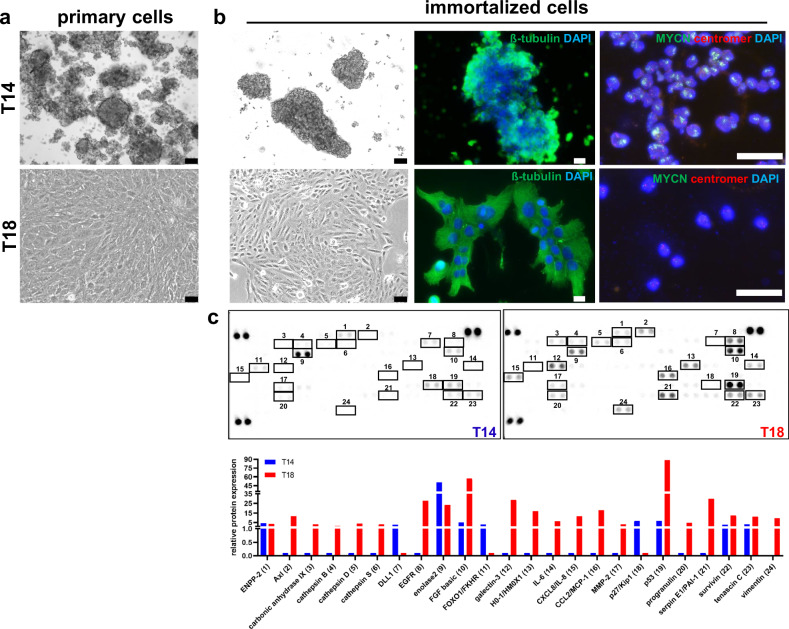
Comparison of primary and immortalized RB (T14) and RB derived stromal cells (T18). **a** Phase contrast imaging (scale bars: 64 µm) of primary RB tumor and RB derived stromal cells. **b** Phase contrast imaging (scale bars: 64 µm), ß-tubulin (green) and DAPI (blue) immunofluorescence staining (scale bars: 10 µm) and MYCN FISH analysis (outermost right row, scale bars: 50 µm) of the immortalized RB tumor and RB derived stromal cells showing the morphology and subclones with MYCN amplification (T14) and non-MYCN amplified T18 stromal cells. **c** Proteome array analyses of 84 human cancer-related proteins differentially expressed in the immortalized T14 RB and T18 stromal cells.

We successfully immortalized one RB tumor cell line (T14) and one RB derived stromal cell line (T18) in RB culture medium (Fig. 4a) by lentiviral transduction of TERT or LargeT, included microsatellite analysis for future cell line authentication and subsequently characterized both of them. The immortalized T14 RB cells consist of subclones with MYCN amplification, whereas, the immortalized T18 stromal cells are not MYCN amplified (Fig. 4a). These results are consistent with the conditions of the original tumors (Supplementary Fig. 2). In a proteome array analysis of 84 human cancer-related proteins we identified several proteins being solely expressed by the T18 stromal cells like cathepsin B, D and S, interleukins, EGFR and MMP-2 as well as progranulin, Axl and PAI-1 (Fig. 4b). In addition, compared to T14 tumor cells considerably higher expression of FGF basic and p53 was detected for. The three proteins DLL1, FOXO1 and p27 were exclusively expressed by the T14 RB tumor cells (Fig. 4b).

Taken together, we successfully immortalized a RB tumor and stromal cell line, displaying a different MYCN status as well as individual expression of cancer-related proteins.

### Characterization of the immortalized RB tumor cell line T14

**Fig. 5:**
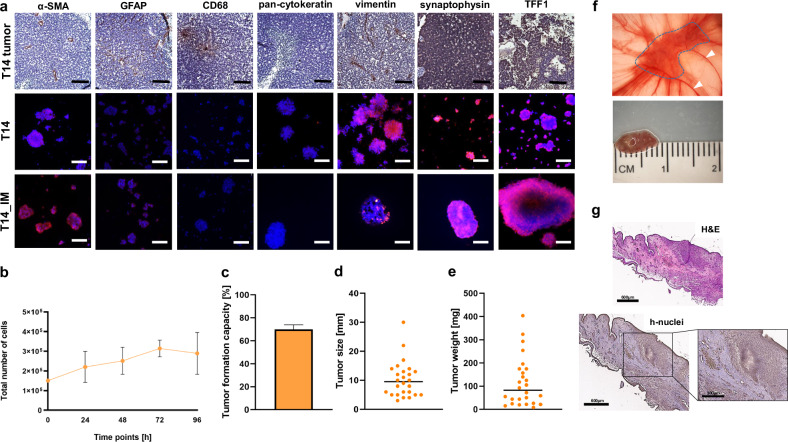
Expression of a set of different markers revealed by immunohistochemical und immunocytochemical stains, growth behavior in vitro and tumor formation in ovo of the newly established, immortalized RB cell line T14. **a** Histological staining of paraffin sections of RB tumor tissue for different markers (brown signal) with hematoxylin and eosin (HE) counterstaining (T14 tumor; upper row) and immunofluorescence staining of primary RB tumorspheres (T14; middle row) and the respective immortalized RB cells (T14_IM; lower row) for indicated markers (red fluorescence) and DAPI counterstaining (blue fluorescence; scale bars: 100 µm). **b** Growth curve analysis of T14_IM RB tumor cells showing mean doubling times of 72 h. **c** Quantification of tumor formation capacity in the chicken chorioallantoic membrane (CAM) model with CAM tumor weight (**d**) and size (**e**). Values are means of three independent experiments. **f** CAM tumor in situ (upper picture) and ruler measurements (in cm) of excised tumors (lower picture) revealing that tumors formed on the upper CAM 7 days after grafting of T14_IM RB cells. Tumor burden is demarcated with a dotted line and arrowheads indicate the main CAM blood vessel. **g** Haematoxylin- eosin (H&E; top) and human anti-nuclei (h-nuclei; bottom) staining of a histological section of a CAM tumor developed after inoculation of T14_IM RB cells. scale bars: 600 µm and 300 µm (zoom in box).

To further characterize the newly established, immortalized primary RB tumor cell line T14 (T14_IM), we used a set of markers including fibroblast (α-SMA), glia (GFAP), tumor associated macrophage (CD68), epithelial (pan-cytokeratin) and mesenchymal (vimentin) markers as well as two RB markers (synaptophysin and TFF1) for immunohistochemical stains. The tumor of origin displayed positive cells for all markers analyzed, except for pan-cytokeratin (Fig. 5a). The primary T14 tumor cells were also strongly positive for the RB markers synaptophysin and TFF1 as well as for vimentin (Fig. 5a). Some α-SMA and GFAP positive cells could also be detected, however, the cells were negative for CD68 and pan-cytokeratin (Fig. 5a). The T14_IM cells, showing a mean doubling time of 72 h (Fig. 5b), likewise reflected the expression pattern of the primary cells. In addition, the T14_IM cells were able to form tumors *in ov*o. The overall tumor formation capacity reached nearly 70% (Fig. 5c) with a mean tumor size of 10 mm (Fig. 5d) and 90 mg (Fig. 5e). Photo documentation and histology of a CAM tumor developing from inoculated T14_IM RB tumor cells showed a CAM tumor surrounded by CAM vessels (Fig. 4f, g).

The immortalized RB tumor cells displayed the maker expression of the original tumor and the primary cells and showed the potential to develop tumors in ovo.

### Characterization of the immortalized RB derived stromal cell line T18

**Fig. 6:**
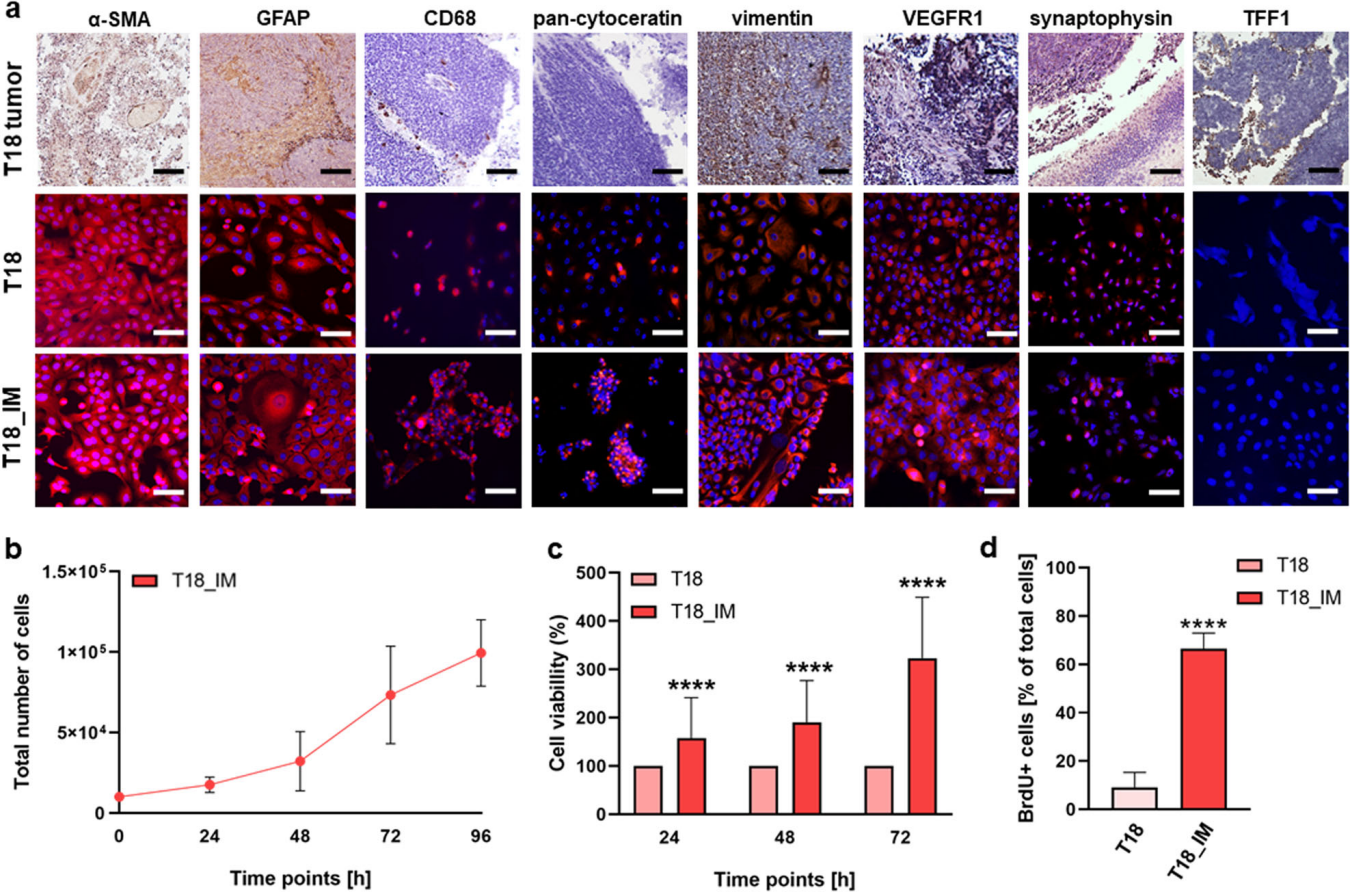
Growth behavior in vitro and expression of different markers of the newly established RB derived stromal cell line T18 as revealed by immunohistochemical stains. **a** Paraffin sections of RB tumor tissue (T18 tumor), primary RB derived stromal cells (T18) and the respective immortalized RB derived stromal cells (T18_IM) stained for different markers (brown signal) with hematoxylin and eosin counterstaining (upper row) or after immunofluorescence staining for markers (red fluorescence) and DAPI counterstaining (blue fluorescence; middle and lower row; scale bars: 100 µm). **b** Growth curve analysis of the immortalized T18_IM RB stromal cells showing mean doubling times of 36 h. Immortalized T18 stromal cells (T18_IM) showed significantly increased cell viability and proliferation levels compared to primary T18 stromal cells (T18) as revealed by (**c**) WST-1 assay and (**d**) BrdU stains. Values are means of three independent experiments ± SEM. ****p < 0.0001 statistical differences compared to the control group calculated by Student’s *t*-test.

We likewise further characterized the newly established, immortalized RB derived stromal cell line T18 (T18_IM). Primary T18 cells stained positive for the stromal cells markers α-SMA, GFAP and CD68 of both, epithelial (pan-cytokeratin) and mesenchymal (vimentin) origin. The tumor of origin displayed positive cells for all markers analyzed, except for pan-cytokeratin (Fig. 6a). The marker set analysis showed that T18_IM cells express all markers analyzed except for TFF1 (Fig. 6a), only expressed by the original tumor. T18_IM stromal cells showed a short mean doubling time of 36 hours (Fig. 6b) and significantly higher cell viability (Fig. 6c) and proliferation levels (Fig. 6d) compared to the primary T18 cells.

Thus, the immortalized stromal cells reflected the marker expression of the tumor of origin as well as of the primary cell culture, but displayed significantly increased proliferation.

### Establishment of a 3D co-cultivation model and MACS separation of RB derived stromal cell populations

**Fig. 7:**
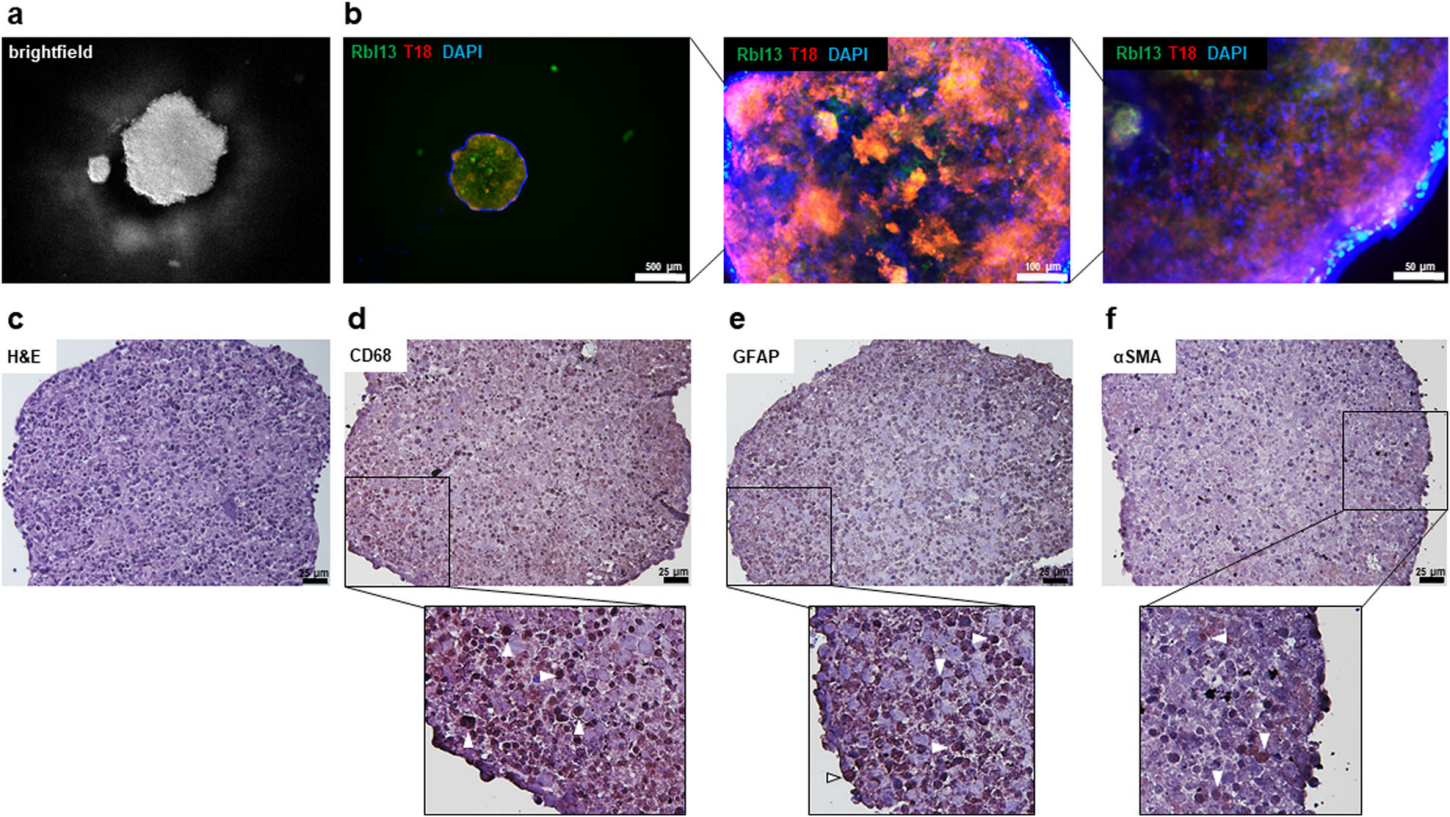
Co-cultivation of RB tumor and stromal cells in a 3D cell culture model. **a** Brightfield and (**b**) immunofluorescence pictures showing Rbl13 tumor cells in green (GFP), T18 stromal cells in red (tomato lectin) and blue nuclei counterstaining (DAPI) of a RB tumor-stroma spheroid in a hanging drop culture at different magnifications (b; scale bars: 500 µm, 100 µm and 50 µm). **c** Histological analysis of paraffin sections of a RB tumor-stroma spheroid stained with different indicated stromal markers and counterstained with hematoxylin and eosin (H&E; **d**–**f**; scale bars: 25 µm). White arrowheads exemplarily indicate some cells, which stained positive for the respective markers (zoomed-in insets **d**–**f**; brown signal).

We established a 3D co-cultivation model for RB tumor and stromal cells based on a hanging drop cultivation method [35], in order to enable future investigations of the interaction of both, stromal and tumor components, on the progression and therapy outcome of RB. Figure 7a depicts a 3D RB stroma-tumor spheroid in the hanging drop cell culture and Fig. 7b displays it’s composition at different magnifications. Both, stromal (T18) and tumor (Rbl13) cell types are uniformly distributed throughout the spheroid as shown by immunofluorescence (Fig. 7b) and stromal marker staining’s of a paraffin embedded spheroid (Fig. 7c-f).

In addition, the different cell populations found in the T18_IM RB derived stromal cell line were successfully separated using MACS separation for tumor-associated macrophages (CD163 + CD68) and glia (GFAP). Thereupon, we re-analyzed the stromal marker set by real-time PCR analysis in order to show the individual expression pattern of each subpopulation (Supplementary Fig. 3a, b). All isolated subpopulations were separately stained for α-SMA, GFAP, CD68, pan-cytokeratin, vimentin, VEGFR1 and the RB marker and TFF1 by immunofluorescence (Supplementary Fig. 3c).

Successful separation of stromal cell populations of the RB TME and establishment of a 3D cell co-culture now enables future investigations of their individual influence on RB tumor cells.

## Discussion

For a personalized RB tumor therapy, it is necessary to understand the intercellular interactions between the tumor and its TME. Several studies analyzing RB tumors at single cell level [12, 30, 36, 37] described the presence of CAFs, TAMs, astrocyte-like cells and few lymphocytes. Investigations on the effect of these TME cell types on tumor development, progression and therapy outcome require an experimental model system including both, RB derived TME stromal and tumor cells. However, to date, only a limited number of RB tumor cell lines [14–16, 38] and as to our knowledge no RB stromal derived cells are available.

Thus, we established primary RB tumor cell cultures from five patients in two cell culture media. Serum-containing medium led to natural separation of suspension RB tumor cells and adherent non-tumor stromal cells, while serum-free medium did not, reflecting previous findings [39]. Sequencing showed RB cells in serum-containing medium to have a more mature phenotype compared to early developmental features observed in serum-free medium. We next set out to immortalize and characterize the RB-derived tumor and stromal cells for long-term studies on the RB-TME interplay. We confirmed tumor and non-tumor origins of the cells by *RB1* mutation, *MYCN* FISH analyses and examined the expression of cancer and stroma-related marker proteins. Furthermore, the comparison of the primary cells with the original tumor tissue showed that the primary tumor cells are very similar to the original tumor, whereas the stromal cells are clearly different. In vivo tumor development of T14 RB tumor cells was verified by CAM assays. In addition, a specific marker panel for RB tumor cells and the RB derived stromal cells was identified and verified.

### Genes included in the RB marker panel

Among the identified RB tumor marker genes are known RB markers like the immature precursor gene CRX as well as genes of mature photoreceptors including phosphodiesterase 6H (PDE6AH) and arrestin 3 (ARR3), specific for cones. In addition spleen tyrosine kinase (SYK) found to be highly expressed in RB tumors [40, 41] and photoreceptor protein recoverin (RCVRN) also expressed in RB [42] were identified as RB tumor cell markers. Furthermore, we identified retina specific interphotoreceptor matrix proteoglycan 2 (IMPG2), phosducin (PDC) and prominin 1 (PROM1) as upregulated genes in the RB tumor group, which are all related to CRX expression in medulloblastoma [43]. PROM1 is known to be expressed in RB cell lines and the adult retina and mutations are associated with cone-rod dystrophy, retinal macular dystrophy and retinitis pigmentosa [44]. Retina and anterior neural fold homeobox 2 (RAX2), TUB like protein 1 (TULP1) and G protein subunit gamma transducin 2 (GNGT2) are associated with photoreceptor cells, with TULP1 being involved in retinitis pigmentosa [45] and RAX2 playing a role in the malignant progression of glioblastoma [46, 47]. In addition, GNGT2 was identified as a potential prognostic marker in esophageal cancer [47] and a hub gene in lung cancer [48]. Crumbs Homolog 1 gene (CRB1) plays an essential role in normal vision and is expressed by the inner segment of photoreceptor cells. Besides, CRB1 chromosome 1q gain was identified as potential driver in retinoblastoma progression [49].

### Genes included in the RB stromal marker panel

Our identified and verified stromal marker set comprises ten of the most upregulated genes found in RB stromal cells in comparison to RB tumor cells. The connective tissue growth factor (CTGF) is expressed upon induction of the transforming growth factor beta (TGFβ) and in turn downregulates p27 [50]. Both CTGF and TGFβ are upregulated in RB derived stromal cells with p27 being downregulated. Furthermore, it has been shown that the migration and angiogenesis factor CTGF was downregulated in retinoblastoma upon verteporfin treatment [51]. Periostin (POSTN) is a secreted extracellular matrix protein, likewise induced by TGFβ and strongly connected to the enhancement of motility, invasion and metastasis of colorectal and ovarian cancer cells [52–55]. POSTN is mainly expressed by CAFs and its expression induces the expression of a disintegrin and metalloproteinase 17 (ADAM17) in esophageal squamous cell carcinoma cells resulting in tumor progression [56]. Interestingly, our group recently demonstrated that ADAM17 is also involved in retinoblastoma tumor progression [57]. In addition, thrombospondin-1 (THBS1), an extracellular matrix protein, is induced by TGFβ and promotes migration and invasion of cancer cells [58]. It is linked to immunosuppression and unfavorable prognosis of colorectal cancer [59]. Along this line it could be shown that fibrillin-1 (FBN1) is a TGFβ modulator and regulates mesenchymal stem cell activity within the microenvironment of marrow niches [60]. Besides, COL1A1 expressed by ovarian cancer fibroblasts promoted migration and invasion of ovarian cancer cells [61] and COL1A2 was identified as a hub node in the stromal module of breast cancer [62]. By contrast, CCDC80 was downregulated in murine gastric cancer fibroblasts and seems to have tumor suppressive functions [63]. The identified genes of both marker sets display a high specificity for RB tumor cells and likewise reflect the supposed influence of TME stromal cells on tumor progression (Supplementary Table 5).

### Known implication of different cells types of the TME on RB tumor growth behavior

Along this line, others could already show that TAMs and astrocyte levels decreased during the invasion process, possibly reflecting an immunosuppressive RB environment and that CAFs may induce RB tumor proliferation [30]. In addition, previous studies also related TAMs to tumor vascularization in cancer [30, 64–66] and invasion of RB tumor cells [30]. In a most recent study [12], macrophages derived from peripheral blood mononuclear cells (PBMC) interacted with soluble factors secreted by retinoblastoma tumor cells, thereby inducing an immunosuppressive RB microenvironment. In accordance, adherent glia from explanted RBs secreted factors that increased proliferation of co-cultured RB cells [28]. In turn, other groups identified differentially expressed genes contributing to RB progression upon RB tumor cell and PBMC co-cultivation [67] or analyzed the influence of RB derived exosomes on macrophages and bone marrow mesenchymal stem cells revealing a pro-tumor effect [68]. Most of these results are, however, based on sequencing analyses, but stillprovided valuable help to decipher intercellular interactions. Nevertheless, it remains necessary to investigate and functionally prove these results by in vitro and in vivo experiments. To address this issue, we separated the heterogeneous RB-derived stromal cell line by MACS to identify individual stromal cell types. Finally, we established a 3D co-culturing system for RB-derived stromal and tumor cells.

In summary, our findings highlight the complexity of retinoblastoma and its TME. The distinct gene expression patterns and morphological characteristics of tumor cells depending on the culture medium, along with the detailed analysis of tumor-stroma interactions, will provide valuable insights into the molecular mechanisms driving tumor growth and progression. The establishment and characterization of primary cultures, immortalized cell lines, and 3D co-culture models lay a solid foundation for future research and new therapeutic approaches. In the long term, these insights could contribute to personalized RB treatment strategies and improve prognosis for this rare but serious disease.

## Methods and materials

### Tumor acquisition

Patient RB tumor samples were obtained from five enucleated eyes (T4, T7, T11, T14, T18) at the University Hospital Essen and used for primary cell cultures and comparative marker expression studies. The Ethics Committee of the Medical Faculty of the University of Duisburg-Essen approved the use of retinoblastoma samples (approval # 14-5836-BO) for research conducted in the course of the study presented and written informed consent was obtained from patients’ relatives or parents. *RB1* mutation analysis was performed at the Department of Human Genetics at the University Hospital Essen and MYCN fluorescence in situ hybridization (FISH) analysis was done by the Institute of Pathology in Kassel, Nordhessen.

### Primary RB cell culture

For primary cell culture, tumor tissue was manually comminuted with a small amount of PBS in a culture dish. After a PBS washing step, the cells were centrifuged at 900 rpm for 5 minutes. Afterwards the RB cell pellet was re-suspended in 500 µl–2 mL/ 24 well RB or MEGM medium. RB medium is comprised of Dulbecco´s modified Eagle´s medium (DMEM; PAN-Biotech, Aidenbach, Germany) supplemented with 15% fetal bovine serum (FBS; PAN-Biotech, Aidenbach, Germany), 100 U penicillin/ml and 100 µg streptomycin/ml (Invitrogen, Darmstadt, Germany), 4 mM L-glutamine (Gibco, Karlsruhe, Germany), 50 µM β-mercaptoethanol (Carl Roth, Karlsruhe, Germany) and 10 µg insulin/ml (PAN-Biotech, Aidenbach, Germany). MEGM medium was purchased (MEGM™ Mammary Epithelial Cell Growth Medium BulletKit, CC-3150, Lonza). RB medium is a commonly used, serum containing growth medium for RB cell cultures, whereas MEGM medium is serum-free and often used for stem cell cultures. Cells were cultivated at 37 °C, 10% CO_2_ and 95% humidity. A suspension cell fraction, naturally segregating from an adherent fraction, was manually separated from the adherent cells by pipetting to obtain split RB tumor spheroid and RB stromal cell cultures. Four human retinoblastoma cell lines (RBL-13, RB 355, Y-79 and WERI-Rb1; formerly provided by H. Stephan; [14, 16, 17, 38]) were cultivated in RB medium for comparison. The RB cell lines used were first tested and authenticated by short tandem repeat (STR) analysis. Subsequently, samples of all tested cells were frozen to ensure access to the tested cells for all experiments. In addition, the RB cell lines were regularly analyzed for their individual *RB1* mutation status. All cell lines were regularly tested for mycoplasma.

### Immortalization of primary RB cell cultures

In order to generate lentiviral particles, 6 × 10^6^ human embryonic kidney cells (HEK293T) were transfected with 6 µg of each of the following plasmid DNAs: (I) packaging vectors pczVSV-G [69] and (II) pCD NL-BH [69] and (III) puc2CL6hTERTcoIPwo for transduction of primary tumor cells or (IV) p2Cl7LargeTwo for transduction of primary stromal cells or with a (V) GFP expressing vector pCL7EGwo or with a (VI) tomato lectin expressing vector p2CL9dTOIPw5 each in the presence of 5 µg polyethyleneimine per ml (PEI, branched, Sigma-Aldrich, St. Louis, Missouri, USA) in DMEM medium. After 24 h the medium was changed to Iscove´s Modified Dulbecco´s medium (IMDM, Pan-Biotech, Aidenbach, Germany) with 10% FBS and 1% penicillin/streptomycin and 72 h after transfection viral supernatants were harvested, filtered (0.45 µm filter) and cryoconserved. Stable transduction was performed as described previously [70] and primary tumor cells were selected with 0.2 µg/ml puromycin for one week.

### Establishment of a 3D spheroid co-culture system

To generate 3D cell spheroids, a suspension with the desired cell density (1 × 10^3^ to 1 × 10^4^ cells per 40 µl droplet) was prepared in RB medium and methylcellulosis (1:1; Carl Roth, Karlsruhe, Germany). The 40 µL droplet suspensions were carefully dispensed onto the lid of a 35 mm Petri dish (Greiner, Leuna, Germany) ensuring that the droplets were spaced adequately to prevent coalescence. The droplets containing lid was gently inverted closing the Petri dish filled with PBS. The droplets were incubated at 37 °C, 10% CO_2_ and 95% humidity for four days, allowing them to aggregate and form spheroidal cell structures within the hanging drops. Thereafter, the cell spheroids within the drops were gently aspirated from each lid and used for further analysis.

### DNA and RNA extraction and quantitative real-time PCR

DNA was isolated with DNeasy Kit (Qiagen, Hilden, Germany) and RNA was isolated using the miRNeasy Kit (Qiagen, Hilden, Germany). For quantitative real-time PCR analyses, cDNA was synthesized with the QuantiTect Reverse Transcription Kit (Qiagen, Hilden, Germany) according to the manufacturer´s protocol. For analysis of tumor and stroma marker expression a SYBR^TM^ green PCR assay (Applied Biosystem, Darmstadt, Germany) was used with specific primers (Supplementary table 1).

### RNA seq analysis

Concentration and quality of RNA was measured with Qubit (Invitrogen, Waltham, MA, USA) and Agilent Bioanalyzer DNA HS (Agilent, Santa Clara, CA, USA). Library preparation was performed with Lexogens QuantSeq 3’ mRNA-Seq Library Prep Kit FWD and sequenced on a NextSeq500 (Illumina, San Diego, CA, USA). Sequences were trimmed with TrimGalore (v.0.6.0 10.14806/ej.17.1.200) and aligned with hisat2 (10.1038/s41587-019-0201-4) to hg38. Statistical analysis was performed with R (R: A language and environment for statistical computing, R foundation for statistical computing, Vienna, Austria, version (v) 4.2.0 2022, https://www.R-project.org/) using the R-packages DESeq2 (10.1186/s13059-014-0550-8), pheatmap (v 1.0.12; Kolde R (2019); pheatmap: pretty heatmaps), umap (v 0.2.8.0; Konopka T (2022); umap: uniform manifold approximation and projection), fgsea(10.1101/060012) and EnhancedVolcano (v 1.14.0; Blighe K, Rana S, Lewis M (2022). EnhancedVolcano: Publication-ready volcano plots with enhanced colouring and labeling).

### Calculation of differentially expressed genes (DEGs)

For the calculation of DEGs, DESeq2 was used. In short: count values were normalized using DESeq2 “Median of ratios” method, which takes sequencing depth and RNA composition into account. For each comparison, all samples of condition 1 and condition 2 were used as biological replicates for DEG analysis. DESeq2 uses a Wald test to test for DEGs applying the null hypothesis “no differential expression across the two sample groups (LFC = = 0)”. False discorvery rate (FDR) was used to correct for the multiple testing problem in bulkRNAseq.

### Cancer-related protein expression profiling

The expression levels of 84 human cancer-related proteins were evaluated in T14 RB tumor and T18 RB derived stromal cells using the Proteome Profiler Human XL Oncology Array (R&D Systems, Minneapolis, MN, USA). The expression levels were determined in duplicates, using 200 μg of protein following the manufacturer’s protocol.

### Cell viability, proliferation and growth kinetic

To determine cell viability, 4 × 10^4^ cells in 100 µl medium were seeded in a 96-well plate in two triplicates. After indicated time points, 10 µl of a water-soluble tetrazolium (WST-1) salt solution (Sigma-Aldrich, München, Germany) was added and the absorbance (450 nm) was measured after incubation at 37 °C for a designated time. Cell proliferation was determined by 5-Bromo-2´-deoxyuridine (BrdU; Sigma, Hamburg, Germany) incorporation as described previously [71]. To determine growth kinetics in a 24-well plate format, 1 × 10^4^ (RB derived stromal cells) or 2 × 10^5^ (RB tumor cells) cells were seeded in 1 ml RB medium in triplicates and cells were counted manually every 24 hours in a Neubauer chamber using the trypan blue exclusion method.

### CAM assays

In order to reveal the capacity of the immortalized RB tumor cells to form tumors *in ovo*, the T14 cells were inoculated on the extraembryonic chorioallantoic membrane (CAM) of chick embryos at embryonic developmental day (EDD)10 mainly following the protocols published by Zijlstra and Palmer [72, 73]. Ten eggs were inoculated with 1 × 10^6^ cells suspended in 50 µl PBS in at least three independent experiments. Seven days after grafting, at EDD17, grown tumors were excised, measured weighted and photographed as described previously [74].

### Immunohistochemistry

For immunofluorescence staining, 1 × 10^5^ RB tumor or 2.5 × 10^4^ RB derived stromal cells, respectively, were seeded on poly-D-lysine (Sigma-Aldrich, München, Germany) coated coverslips and processed as described previously [71]. The Vectastain Elite ABC kit (Biozol, Eching, Germany) was used for immunohistochemical stainings of formalin fixed, paraffin embedded retinoblastoma sample sections as described previously by our group [57]. The reaction was visualized by 3,3´-diaminobenzidine (DAB; Sigma-Aldrich, München, Germany) staining and the sections were counterstained with haematoxylin. Antibodies and concentrations used are listed in Supplementary table 2.

### Magnetic cell separation

We used the MiniMACS™ Starting Kit (Miltenyi Biotec, Bergisch Gladbach, Germany) for magnetic cell separation of 1 × 10^7^ immortalized RB derived stromal cells (T18_IM) with the following antibodies included in the kit: GFAP (130-118-489), CD163 (130-112-286), and CD68 (130-114-651) that were bound by Anti-PE MicroBeads (130-048-801). Cell separation was done according to the manufacturer’s protocol.

### Statistical Analysis

All assays were performed at least in triplicates. Statistical analyses were performed using GraphPad Prism 9. Data represent means ± SEM of three independent experiments from independent RB cell cultures. Results were analyzed by a Student’s *t*-test and considered significantly different if *p*-value < 0.05 (*), *p*-value < 0.01 (**), *p*-value < 0.001 (***) or *p*-value < 0.0001 (****).

## Supplementary information

Supplementary material

## Data Availability

The RNAseq data used to support the findings of this study have been deposited in the Gene Expression Omnibus (GEO) database (GSE274663).
