# Supplementary material for: In vitro model of retinoblastoma derived tumor and stromal cells for tumor microenvironment (TME) studies
Source: Cell Death Dis. 2024 Dec 18;15(12):905. doi: 10.1038/s41419-024-07285-2 (PMC11655973; doi:10.1038/s41419-024-07285-2)
Supplement: Supplementary file 1 — Supplementary material [file 41419_2024_7285_MOESM1_ESM.pdf]

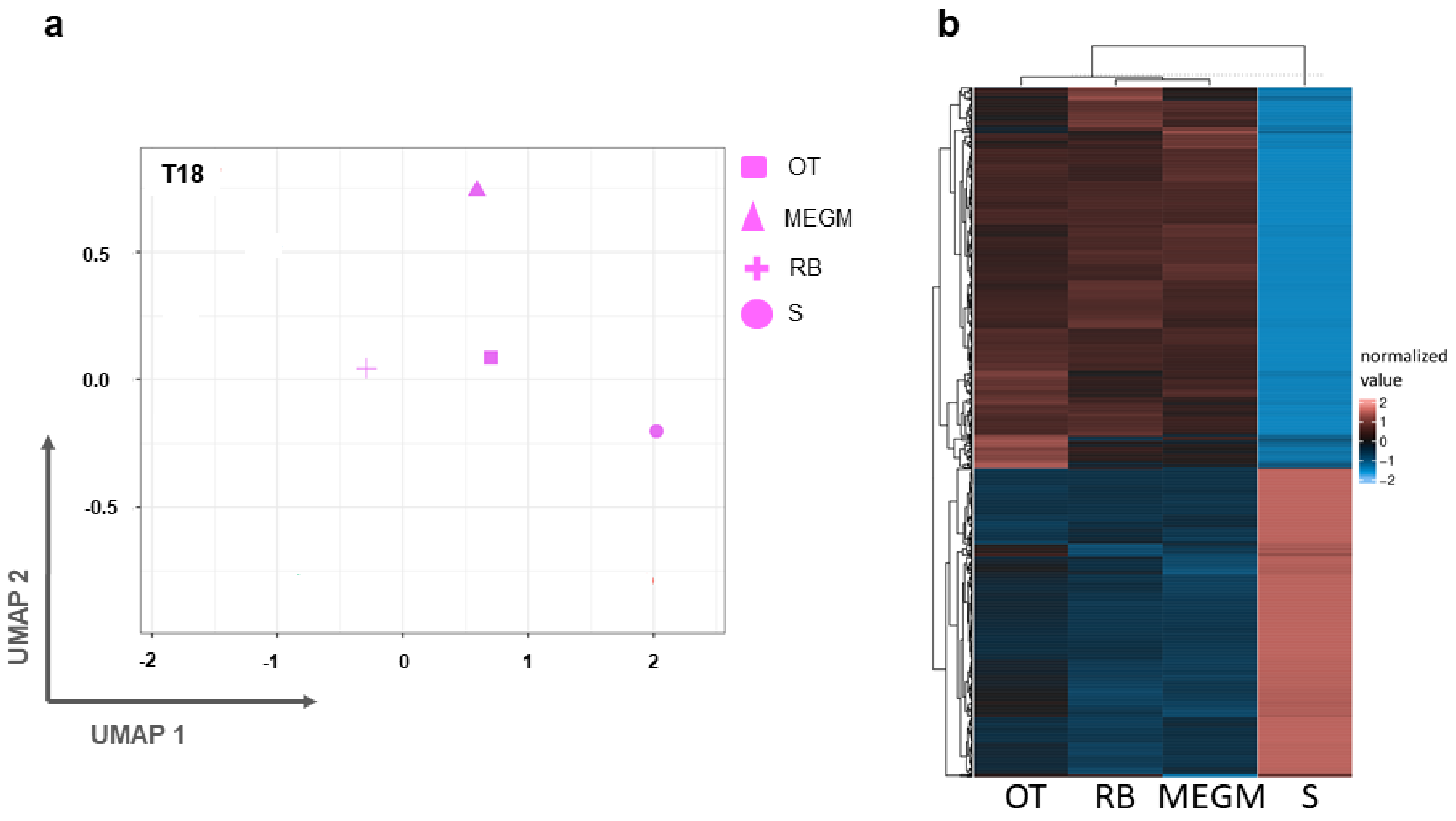

Supplementary Figure 1. UMAP and heatmap analysis of T18. (a) UMAP analysis of the original tumor (OT), primary RB cells in MEGM and RB medium and RB derived stromal cells (S) of T18 displayed a separation of the stromal cells. (b) Heatmap of significantly up- and down-regulated DEGs with a minimum fold change of 1.5.

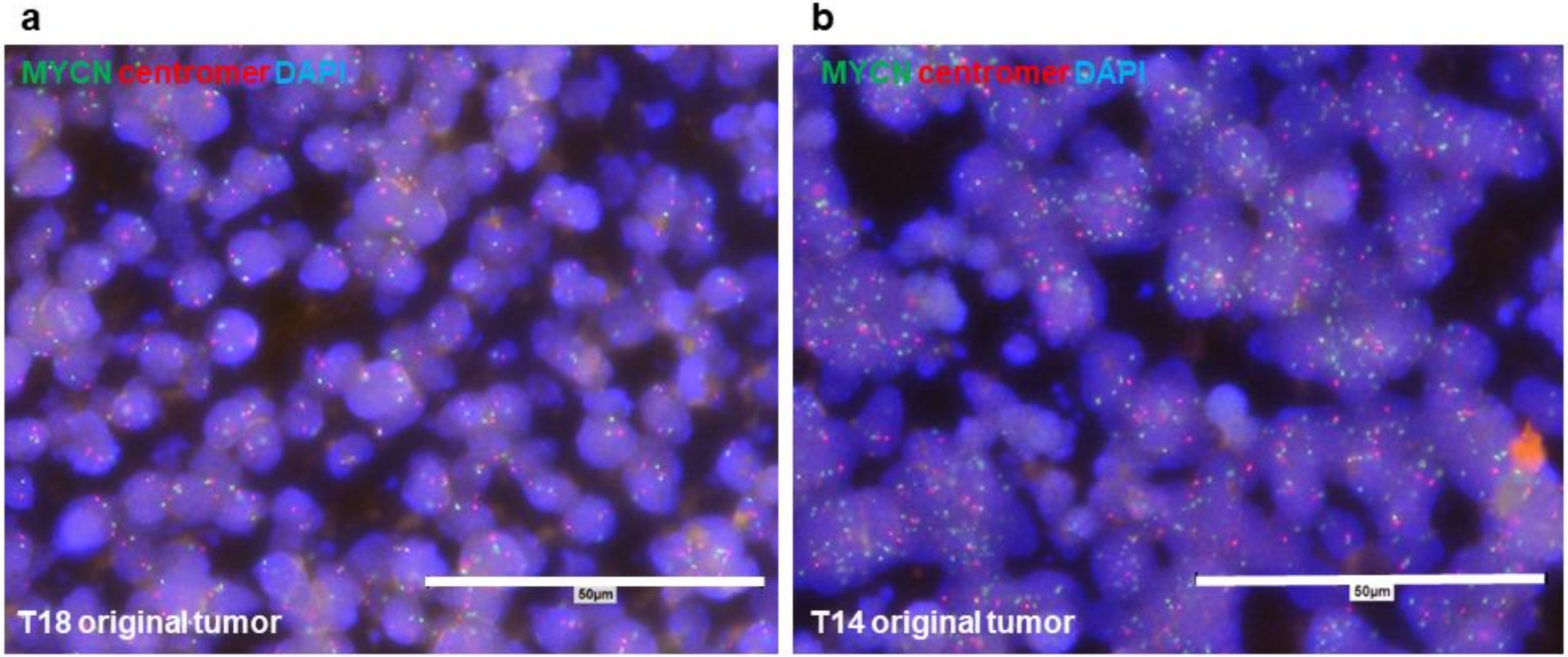

Supplementary Figure 2. MYCN FISH analysis of the original RB tumor T18 without MYCN amplification (a) and MYCN amplified T14 original RB tumor (b) (scale bars: 50µm).

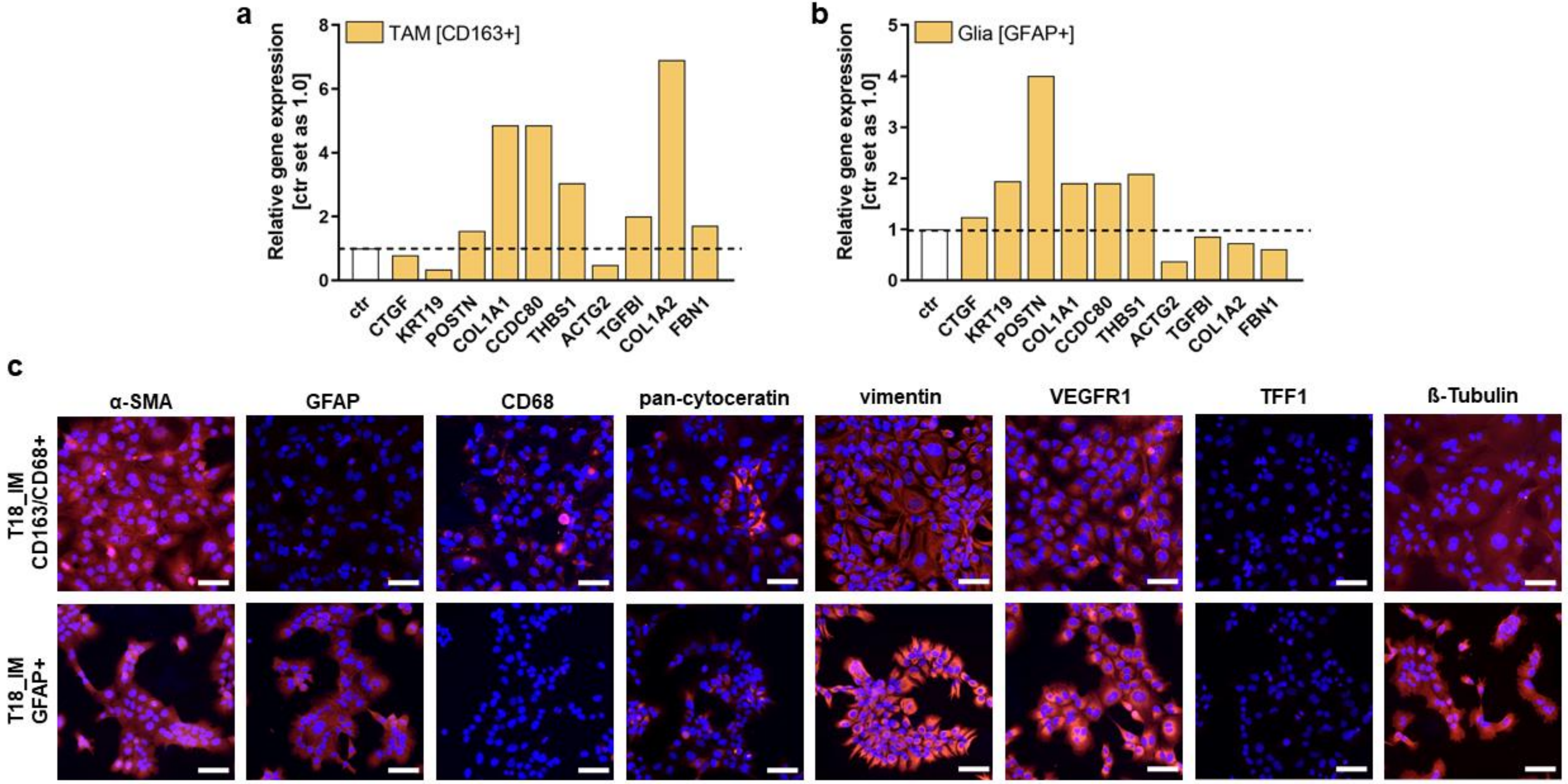

Supplementary Figure 3. Relative expression levels of genes defined as the "stromal marker set" analyzed in MACS separated stromal subpopulations of TAMs (a) and glia (b) in comparison to the whole RB derived stromal cell population of T18_IM cells (ctr; white columns) as revealed by real-time PCR. (c) Immunofluorescence staining for different markers (red fluorescence) and DAPI counterstaining (blue fluorescence; scale bars: 100 µm) of MACS separated stromal subpopulations of TAMs (CD163/CD68+) and glia (GFAP+).

Supplementary table 1. Primer sequences for the genes analyzed by real-time PCR analysis. FW: forward primer, RV: reverse primer

**Gene**	**Primer sequences (FW and RV)**
CRX	5´-CTCAGTGTCCCCGAAGATCA-3´
	5´-GGTACTGGGTCTTGGCAAAC-3´
GAPDH	5´- ACCCACTCCTCCACCTTTGA-3´
	5´-CTGTTGCTGTAGCCAAATTCGT-3`
SYK	5´-CCGTATGAGCCAGAACTTGC-3´
	5´-ACCAGAGCCCAGTTCTTTGT-3´
IMPG2	5´-TGGAAAACCATGGCCTTGTG-3´
	5´-GCGTTGCCTGAAGACTTGAA-3´
RCVRN	5´-CGGGACCATCAGCAAGAATG-3´
	5´-CCAAGCACAGAGGGTCATTG-3´
ARR3	5´-CGGGAATGGACAAAGAGCTG-3´
	5´-CTTTCCGCGTAAACTCCTCG-3´
PDC	5´-ACCAGGCACAGAGATCCAAA-3´
	5´-CTGAGGAGAAGACATTTGCCTG-3´
TULP1	5´-CCGTCATCATTCCTGGCATG-3´
	5´-CAGCGTGGACAATCTGGAAG-3´
GNGT2	5´-CTGTGTCTCTCTGTCCCCTG-3´
	5´-GCCCTAGACAGACCTGATCC-3´
PDE6H	5´-ACAACACTACTCTGCCTGCT-3´
	5´-GCCTCCCATGGACAAATCAC-3´
CRB1	5´-AATGTAACCCAAGGCTGTGC-3´
	5´-ATCCACACCACTGAACCTCC-3´
RAX2	5´-ACTACCCGGATGTGTACAGC-3´
	5´-TCGGGGAGTCTCGGAGCT-3´
PROM1	5´-TTCTTGACCGACTGAGACCC-3´
	5´-CCAAGCACAGAGGGTCATTG-3´
CTGF	5´-TACCAATGACAACGCCTCCT-3´
	5´-CCGTCGGTACATACTCCACA-3´
KRT19	5´-CGCGACTACAGCCACTACTA-3´
	5´-AGAGCCTGTTCCGTCTCAAA-3´
POSTN	5´-CCTTGGAAGAGACGGTCACT-3´
	5´-CTCAAAGACTGCTCCTCCCA-3´
COL1A1	5´-GCTACTACCGGGCTGATGAT-3´
	5´-ACCAGTCTCCATGTTGCAGA-3´
CCDC80	5´-TCTGATCACTGCTCCCAAGG-3´
	5´-CATCATCCACCACTCGCATG-3´
THBS1	5´-GCAAGTCACCCAGTCCTACT-3´
	5´-AATGAAACCCGTCTTTGGCC-3´
ACTG2	5´-CTGGCACCACTCCTTCTACA-3´
	5´-CAGAGGCATAGAGGGAGAGC-3´
TGFBI	5´-CCTGAGAGACCTGCTGAACA-3´
	5´-GTCTTGGCTGAGTCTGGGAT-3´
COL1A2	5´-TAGAAAGAACCCAGCTCGCA-3´
	5´-GGGATGTTTTCAGGTTGGGC-3´
FBN1	5´-GACATCAATCTGTGCGGGTC-3´
	5´-CAACACACTGGTTCCACTGG-3´

Supplementary table 2. Antibodies used for immunofluorescence (IF) and immunohistochemical (IHC) stainings.

Antibody	Clone	Dilution (IF)	Dilution (IHC)	Buffer (IHC)
anti-alpha smooth muscle actin antibody	ab5694 (abcam)	1:100	1:1000	EDTA buffer (pH9)
GFAP	ASTRO6 (Invitrogen)	1:100	1:500	EDTA buffer (pH9)
CD68	KP1 (Invitrogen)	1:200	1:500	EDTAbuffer (pH9)
synaptophysin	YE269 (abcam)	1:100	1:600	citrate buffer (pH6)
pan-cytokeratin	Sc-8018 (Santa Cruz)	1:100	1:100	EDTA buffer (pH9)
vimentin	Sc-6260 (Santa Cruz)	1:100	1:100	EDTA buffer (pH9)
VEGFR1	PA5-32408 (Invitrogen)	1:100	1:200	Citrate buffer (pH6)
TFF1	ab92377 (abcam)	1:200	1:250	Citrate buffer (pH6)
antinuclei	MAB1281 (Merck Millipore)	-	1:100	Citrate buffer (pH6)
β-Tubulin	TUB 2.1 (Sigma Aldrich)	1:200	-	-

Supplementary table 3. Pathway gene enrichment analysis with the DEG selection of tumorspheres grown either in RB medium or MEGM medium. Shown are up to ten of the most significant GO terms and KEGG pathways analized by DAVID.

Go term category	Tumorspheres in RB medium		Tumorspheres in MEGM medium	
**Biological process (BP)**	**Term**	**P-Value**	**Term**	**P-Value**
	visual perception	0.0000077	axon guidance	0.0005
	cell adhesion	0.0014	kindey development	0.0021
	phototransduction	0.0018	negative chemotaxis	0.0028
	membrane repolarization	0.0032	response to xenobiotic stimulus	0.013
	regulation of G-protein coupled receptor protein signaling pathway	0.006	brain development	0.017
	retina development in camera-type eye	0.011	response to drug	0.023
	positive regulation of Fc receptor mediated stimulators signaling pathway	0.016	neuron projection development	0.027
	retina layer formation	0.018	neuron cell-cell adhesion	0.035
	transmembrane receptor tyrosine kinase signaling pathway	0.021	cellular response to organic substance	0.035
	regulation of ERK1 and ERK2 cascade	0.022		
**Cell composition (CC)**	**Term**	**P-Value**	**Term**	**P-Value**
	photoreceptor inner segment	0.0014	integral component of membrane	0.0032
	dentrite membrane	0.0016	plasma membrane	0.0073
	photoreceptor outer segment	0.0019	cell surface	0.013
	synapse	0.011		
	lamelliopodium membrane	0.012		
	focal adhesion	0.015		
	plasma membrane	0.016		
	dendric branch	0.023		
	cell surface	0.023		
	cell junction	0.03		
**Molecular function (MF)**	**Term**	**P-Value**	**Term**	**P-Value**
	ion channel binding	0.0044	chemorepellent activity	0.0018
	protein tyrosine kinase activity	0.01	semaphorin receptor binding	0.049
	histone demethylase activity	0.013		
	chemorepellent activity	0.021		
	binding, bridging	0.017		
	integrin binding	0.035		
**KEGG pathways**	**Category**	**P-Value**	**Category**	**P-Value**
	phosphatidylinositol signaling system	0.0082	axon guidance	0.0041
	proximal tubule bicarbonate reclamation	0.015		
	thyroid hormone signaling pathway	0.017		
	inositol phosphate metabolism	0.022		
	arrhythmogenic right ventricular cardiomyopathy	0.025		
	PI3K-Akt signaling pathway	0.026		
	ECM-receptor interaction	0.036		
	GABAeric synapse	0.037		
	AGE-RAGE signaling pathway in diabetic complications	0.049		

Supplementary table 4. Pathway gene enrichment analysis with the DEG selection of stroma-like RB derived cells and tumorspheres both grown in RB medium. Shown are up to ten of the most significant GO terms and related diseases analzed by DAVID.

GO term category	Stroma-like RB derived cells		Tumorspheres in RB medium	
**Biological process (BP)**	**Term**	**P-Value**	**Term**	**P-Value**
	angiogenesis	1.3 E-24	visual perception	2.4 E-21
	positive regulation of cell migration	1.9 E-23	chemical synaptic transmission	2.5 E-20
	cell adhesion	1.1 E-19	potassium ion transmembrane transport	5.1 E-14
	collagen fibril organisation	1.5 E17	nervous system development	6.7 E-14
	cell migration	2.5 E-17	regulation of ion transmembrane transport	1.1 E-12
	wound healing	2.5 E-16	regulation of membrane potential	3.1 E-12
	positive regulation of gene expression	5.8 E-15	axon guidance	1.5 E-9
	extracellular matrix organization	3.9 E-14	brain development	2.8 E-9
	signal transduction	2.7 E-13	synapse assembly	4.9 E-9
	negative regulation of apoptotic process	6.9 E-12	retina development in camera-type eyes	7.0 E-6
**Cell composition (CC)**	**Term**	**P-Value**	**Term**	**P-Value**
	extracellular exosome	2.9 E-73	photoreceptor inner segment	1.6 E-23
	focal adhesion	2.1 E-59	glutamatergic synapse	1.1 E-22
	endoplasmatic reticulum lumen	5.2 E-33	synapse assembly	5.1 E-22
	cytosol	6.4 E-27	axon guidance	2.0 E-21
	extracellular region	6.4 E-27	neuron projection	2.2 E-21
	cell surface	6.9 E-26	dendrite	1.8 E-18
	membrane	7.2 E-25	postsynaptic density	1.1 E-17
	plasma membrane	3.6 E-24	neuronal cell body	5.5 E-17
	extracellular matrix	1.7 E-23	dendric spine	9.8 E-14
	golgi apparatus	2.3 E-21	photoreceptor outer segment	2.0 E-12
**Molecular function (MF)**	**Term**	**P-Value**	**Term**	**P-Value**
	protein binding	1.5 E-32	voltage-gated potassium channel activity	1.0 E-12
	extracellular matrix structural constituent	4.3 E-22	calmodulin binding	8.1 E-10
	cadherin binding	4.9 E-19	microtubule binding	1.9 E-9
	integrin binding	5.7 E-19	calcium ion binding	1.6 E-8
	collagen binding	1.3 E-15	ligand-gated ion channel activity	8.3 E-7
	actin binding	1.4 E-14	ATP-dependent microtubule motor activity	7.2 E-6
	identical protein binding	2.6 E-13	delayed rectifier potassium channel ctivity	8.5 E-6
	protein homodimerization activity	3.0 E-13	GTPase activator activity	1.4 E-5
	calcium ion binding	9.5 E-13	3´,5´-cyclic-nucleotide phosphodiesterase activity	2.1 E-5
	actin filament binding	2.0 E-10	potassium channel activity	3.6 E-5
**Disease**	**Term**	**P-Value**	**Term**	**P-Value**
			retinitis pigmentosa	4.4 E-7
			cone-rod dystrophy	1.3 E-5
			congenital stationary night blindness	1.9 E-2

Supplementary table 5. RB tumor and stromal marker expression.

RB tumor marker set	RB tumorspheres	RB cell lines
**CRX**	expressed	expressed
**SYK**	expressed	expressed
**IMPG2**	expressed	expressed
**RCVRN**	expressed	expressed
**ARR3**	expressed	expressed
**PDC**	expressed	expressed
**TULP1**	expressed	expressed
**GNGT2**	expressed	not expressed
**PDE6H**	expressed	expressed
**CRB1**	expressed	not expressed
**RAX2**	expressed	not expressed
**PROM1**	expressed	expressed

RB stromal marker set	RB stromal cells	TAM [CD163+]*	Glia [GFAP+]*	RB cell lines
**CTGF**	expressed	decreased	increased	not expressed
**KRT19**	expressed	decreased	increased	not expressed
**POSTN**	expressed	increased	increased	not expressed
**COL1A1**	expressed	increased	increased	expressed
**CCDC80**	expressed	increased	increased	not expressed
**THBS1**	expressed	increased	increased	not expressed
**ACTG2**	expressed	decreased	decreased	not expressed
**TGFBI**	expressed	increased	decreased	not expressed
**COL1A2**	expressed	increased	decreased	not expressed
**FBN1**	expressed	increased	decreased	not expressed

*compared to bulk RB stromal cells
